# Exploring the relationship of complementary therapy use and medication adherence among patients with epilepsy

**DOI:** 10.1080/20523211.2025.2540786

**Published:** 2025-08-05

**Authors:** Siti Nor Aqilah Mohd Noor, Nurul Afiedia Roslim, Shazia Jamshed, Chiau Ming Long, Umar Idris Ibrahim, Ahmad Kamal Ariffin Abdul Jamil, Nurulumi Ahmad, Aslinda Jamil, Kheng Seang Lim, Mazlina Husin, Khairul Azmi Ibrahim, Norsima Nazifah Sidek, Sulaila Basiam, Saidatul Manera Mohd Daud, Rose Izura Abdul Hamid, Pei Lin Lua

**Affiliations:** a Faculty of Pharmacy, Universiti Sultan Zainal Abidin (UniSZA), Kampus Besut, Besut, Malaysia; b Cawangan Taman Laut & Pengurusan Sumber Daerah Besut, Jabatan Perikanan Negeri Terengganu, Besut, Malaysia; c School of Pharmacy, International Medical University (IMU), Kuala Lumpur, Malaysia; d School of Medical and Life Sciences, Sunway University, Selangor, Malaysia; e Faculty of Medicine, University of Malaya, Kuala Lumpur, Malaysia; f Hospital Sultanah Nur Zahirah, Kuala Terengganu, Malaysia; g Hospital Tengku Ampuan Afzan, Kuantan, Malaysia; h Hospital Raja Perempuan Zainab II, Kota Bharu, Malaysia

**Keywords:** Epilepsy, complementary and alternative therapies, medication adherence, antiepileptic drugs, knowledge, attitudes and practices

## Abstract

**Background:**

Complementary and alternative therapies (CATs) are widely used among patients with epilepsy (PWE), yet their impact on adherence to antiepileptic drugs (AEDs) remains limited and unclear. This study aimed to (i) assess the usage, knowledge, attitudes and practices (KAP) related to CATs and (ii) evaluate their impact on AEDs adherence among PWE.

**Methods:**

A cross-sectional study was conducted among 193 PWE, recruited conveniently from three hospitals on the East Coast of Peninsular Malaysia. Participants’ data were collected through face-to-face administration of paper-based questionnaires, including a KAP-CATs and the Malaysia Medication Adherence Scale (MALMAS). The SPSS version 26.0 was used to perform descriptive statistics and non-parametric tests.

**Results:**

Among the participants (mean age = 35.3 ± 12.6 years; female = 54.9%; Malay = 96.9%), 59.6% reported had used some types of CATs, with prayers (86.4%) and massage (78.8%) being the most common. Most participants demonstrated moderate knowledge (61.6%), neutral attitudes (78.6%) and a moderate engagement in CATs practices (47.6%). Common reasons for using CATs included greater availability (54.9%), belief in self-healing (45.6%) and the perception of a permanent cure (44.6%). Notably, only 28.5% of CATs users disclosed their use to a healthcare provider. The prevalence of non-adherence to AEDs was 22.8%, and no statistically significant association was found between usage, knowledge, attitude and practice regarding CATs and non-adherence to AEDs (*p* > 0.05).

**Conclusion:**

The findings highlight widespread use of CATs among PWE, often driven by cultural beliefs and perceived benefits. Thus, further research is warranted to explore integrative care models that ensure safe, coordinated epilepsy management.

## Background

Epilepsy is a chronic neurological disorder that requires long-term adherence to antiepileptic drugs (AEDs) to maintain seizure control and prevent complications (Fisher et al., 2005). Despite the availability of medications, non-adherence remains a global concern, often leading to poor health outcomes, increased hospitalisation rates and reduced quality of life (Donahue et al., 2025; Singh et al., 2024). In response to the limitations of conventional treatment, such as medication side effects, suboptimal seizure control and psychosocial factors, many patients with epilepsy (PWE) turn to complementary and alternative therapies (CATs) as adjunctive or substitute approaches. According to the National Centre for Complementary and Integrative Health (NCCIH) (2021), CATs encompass a broad range of non-conventional interventions, including herbal remedies, dietary modifications, traditional medicine and spiritual practices.

The global use of CATs is extensive and often shaped by cultural beliefs, personal experiences and the perceived naturalness or safety of these therapies (Romero-García et al., 2024). For example, herbal therapies are commonly used by individuals with diabetes to manage blood glucose levels, while acupuncture has been applied to address diabetic neuropathy (Cho & Kim, 2021; Haxhiraj et al., 2024; Krawinkel et al., 2018). Among cancer patients, aromatherapy is frequently used to alleviate chemotherapy-related symptoms (Hudiyawati et al., 2022; Lua & Zakaria, 2012), and stress-reduction practices such as yoga and relaxation techniques are increasingly adopted by those with hypertension, depression and anxiety (Prasad, 2024; Rajagopalan et al., 2022). The CATs usage among PWE varies widely, with prevalence rates ranging from 7.5% to 73.3% across different populations and regions (Farrukh et al., 2021; Koh et al., 2022; Zhao et al., 2022). In the context of epilepsy, CATs use is often driven by dissatisfaction with conventional AEDs, including concerns about limited efficacy, undesirable side effects and the perception that CATs offer a more holistic solution (Can et al., 2024; Koh et al., 2022). Besides, cultural beliefs and community influence further shape positive attitudes towards CATs, prompting individuals to incorporate these therapies in their treatment regimens (Farrukh et al., 2021; Koh et al., 2022). While some CATs modalities may offer adjunctive benefits, such as improved seizure control or quality of life, scientific evidence supporting their effectiveness remains inconsistent.

In addition, the role of CATs in influencing AEDs adherence is not well understood. While some studies suggest CATs use may coexist with conventional treatment, others indicate it could lead to reduced medication adherence due to misconceptions or conflicting health beliefs (Koh et al., 2022; Singh et al., 2024). The reported non-adherence rates among PWE who use CATs range between 25% and 68%, reflecting a potential significant clinical concern as it may lead to increased seizure frequency (Jopowicz et al., 2024; Singh et al., 2024). Despite growing interest in CATs among PWE globally, limited research has explored how CATs use intersects with adherence behaviours. Moreover, inadequate knowledge about CATs may lead to unsafe practices, including drug interactions or delayed medical care, which can negatively impact treatment outcomes. Therefore, this study aims to assess the usage, knowledge, attitudes and practices (KAP) related to CATs and evaluate the relationship between CATs use and AEDs adherence among PWE in the East Coast of Peninsular Malaysia.

## Methods

### Study design and setting

This cross-sectional study was conducted in three hospitals located on the East Coast of Peninsular Malaysia: Hospital Sultanah Nur Zahirah (HSNZ) in Terengganu, Hospital Tengku Ampuan Afzan (HTAA) in Pahang, and Hospital Raja Perempuan Zainab II (HRPZ II) in Kelantan. The study was carried out from August 2023 to July 2024.

### Sample size and participants

The sample size was calculated using the Power and Sample Size (PS) software version 3.1.2, based on the standard deviation from a prior study by Farrukh et al. (2021), a 5% margin of error, 95% confidence level and 50% response distribution. A sample size of 193 was determined to be sufficient. Participants were recruited using convenience sampling. Convenience sampling was employed due to the practical constraints of recruitment in the outpatient clinic settings, whereby patients were less likely to remain for long during visits. Additionally, the three recruitment centre are the largest epilepsy clinics in public hospitals in East Coast Peninsular Malaysia. Individuals who were at least 18 years old, had a confirmed diagnosis with any type of epilepsy, and were able to communicate in the Malay or English language were included in this study. Participants were excluded if their health or behaviour, including cognitive impairment, and speaking difficulties, posed challenges that may interfere with the data collection process.

### Study instruments

The questionnaire comprised of three main sections, which were adopted from previous studies (Chung et al., 2015; Farrukh et al., 2021). Section A gathered data on the participants’ sociodemographic such as age, gender, ethnicity, marital status, education level, monthly income and employment status. Section B covered the types of CATs and their usage. Section C consisted of knowledge (4 items) with a correct answer scored as one, attitude (8 items) and practice of CATs (8 items) (Farrukh et al., 2021). Section D consisted of the Malaysian Medication Adherence Scale (MALMAS) to assess adherence, with a score of 6–8 indicates adherence, while a score below 6 indicates non-adherence to AEDs (Chung et al., 2015).

For the knowledge component, each item had two response options: ‘yes’ or ‘no’, with correct answers score of 1 and incorrect as 0. Eight questions of attitude, scored as follows: Strongly agree = 5, Agree = 4, Not sure = 3, Disagree = 2, and Strongly disagree = 1; however, one item (Question 8) was reverse scored to account for its negative phrasing, ensuring consistency in the direction of the scale**.** The practice component consisted of six items, scored in the reverse order: Strongly agree = 1, Agree = 2, Not sure = 3, Disagree = 4, and Strongly disagree = 5. Two items (Questions 3 and 5) in this section were reverse scored because they were phrased negatively, and reverse scoring helped maintain interpretive consistency in measuring positive practice behaviours. The mean score for each domain (knowledge, attitude, and practice) was calculated across all participants. Participants who scored equal to or above the mean were classified as having ‘good knowledge,’ ‘positive attitude,’ and ‘good practice,’ respectively. Those who scored below the mean were categorised as having ‘poor knowledge,’ ‘negative attitude,’ and ‘poor practice.’ This method is consistent with the approach used in previous studies (Farrukh et al., 2021).

### Data collection procedure

Upon recruitment, the participants were provided with a brief explanation about the study and consent form. All participants were assured that their personal details would remain anonymous, kept confidential and only used for this research. Participants then proceeded to complete a set of questionnaire on sociodemographic characteristics, KAP related to CATs and adherence to AEDs. A token of appreciation was distributed to the participants at the end of the study. All data were collected via face-to-face, paper-based questionnaires during participants’ routine outpatient clinic visits.

### Data analysis

The collected data were analysed using IBM Statistical Package for Social Sciences (SPSS) version 26.0 (Armonk, NY, US). Descriptive statistics, including means, standard deviations, frequencies, and percentages, were used to summarise the sociodemographic data and responses regarding the utilisation and KAP of CATs. Univariate analysis to study the relationship between the CATs usage and adherence to AEDs was done using Chi-square test and independent t-test where necessary. The value of *p* < 0.05 was considered statistically significant.

## Results

### Sociodemographic characteristics

Overall, there were 193 PWE had involved in this study, with most of them were from HSNZ (83.9%), then HRPZ (12.4%), and HTAA (3.6%). The majority of the participants were female (54.9%), Malay (96.9%) and were unmarried (53.4%). The mean age of the participants was 35.3 ± 12.6 years old. The majority had completed secondary education (49.7%), while 37.8% had tertiary-level education. Besides, 82.7% of participants reported monthly income was below RM3,000. Table 1 presents the sociodemographic characteristics of the participants.

**Table 1.** Sociodemographic characteristics of participants (*n* = 193).

Variables	*n* (%)
Location	
HSNZ	162 (83.9)
HRPZ II	24 (12.4)
HTAA	7 (3.6)
Age (mean ± SD)	35.3 ± 12.6
Gender	
Male	87 (45.1)
Female	106 (54.9)
Race	
Malay	187 (96.9)
Chinese	6 (3.1)
Religion	
Islam	187 (96.9)
Buddha	6 (3.1)
Marital status	
Single	103 (53.4)
Married	85 (44.0)
Divorces	5 (2.6)
Educational level	
No formal education	3 (1.6)
Primary education	21 (10.9)
Secondary education	96 (49.7)
Tertiary education	73 (37.8)
Household income level	
Less than RM1,000	70 (37.8)
RM1,000 – RM2,999	83 (44.9)
RM3,000 – RM4,999	15 (8.1)
RM5,000 – RM6,999	9 (4.7)
RM7,000 – RM8,999	4 (2.2)
RM9,000 – RM10,000	1 (0.5)
More than RM10,000	3 (1.6)
Employment status	
Employed	68; 35.2
Unemployed	57; 29.5
Retiree	7; 3.6
Housewife	36; 18.8
Student	25; 13.0

### Usage, knowledge, attitudes and practices of CATs

From Table 2, 59.6% (n = 115) reported had used some types of CATs with the most used CATs among participants were prayers (86.4%), followed by massage (78.8%). Participants generally have a neutral or mildly positive attitude towards CATs, with 42.9% use only CATs, and 52.9% used CATs in combination with modern medicine. Many agreed that CATs are easily available (56.4%), promote self-healing (46.6%) and provide a permanent cure (45.5%). However, not many strongly believe in CATs as a complete replacement, and only a few (29.1%) of the participants communicate this usage to healthcare professionals (Table 3).

**Table 2.** CATs utilisation patterns among participants (*n* = 193).

Item (s)	*n (%)
CATs usage	
Yes	115 (59.6)
No	78 (40.4)
Type of CATs used:	
Acupuncture	5 (4.2)
Aromatherapy	21 (17.8)
Cupping	38 (32.2)
Massage	93 (78.8)
Prayers	102 (86.4)
Structured exercise (aerobics, Zumba, etc)	48 (40.7)
Antioxidants capsule	3 (2.5)
Detoxifying diet	11 (9.3)
Ginseng	2 (1.7)
Spirulina	1 (0.8)
Unknown herbal	6 (5.1)
Vitamins & minerals	51 (43.2)
Homeopathy	20 (16.9)
Traditional Chinese medicine	2 (1.7)
Traditional Indian medicine	2 (1.7)
Traditional Malay medicine	42 (35.6)

*n = frequency.

**Table 3.** Attitude and practice towards CATs (*n* = 189).

Item (s)	Frequency n (%)				
	Strongly agree	Agree	Neutral	Disagree	Strongly disagree
***Attitude***					
CATs … .					
• Provides quick and additional relief.	8 (4.2)	39 (20.6)	84 (44.4)	45 (23.8)	13 (6.9)
• Cheaper compared to modern medicine.	5 (2.7)	39 (20.7)	75 (39.9)	55 (29.3)	14 (7.4)
• Provides a permanent cure.	7 (3.7)	79 (41.8)	73 (38.6)	20 (10.6)	10 (5.3)
• Is easily available	13 (6.9)	93 (49.5)	59 (31.4)	19 (10.1)	4 (2.1)
• Providers give good information about CATs.	9 (4.8)	62 (32.8)	81 (42.9)	33 (17.5)	4 (2.1)
• Promotes self-healing.	9 (4.8)	79 (41.8)	71 (37.6)	22 (11.6)	8 (4.2)
• Should be used only in minor illness.	4 (2.1)	66 (34.9)	74 (39.2)	39 (20.6)	6 (3.2)
• People use CATs due to fearing discomfort from allopathic medicine.	8 (4.2)	68 (36.0)	69 (36.5)	35 (18.5)	9 (4.8)
***Practice***					
• Use CATs because allopathic medicine is more expensive.	2 (1.1)	20 (10.6)	70 (37.0)	76 (40.2)	21 (11.1)
• Use CATs because I have more faith in CATs.	1 (0.5)	17 (9.0)	73 (38.6)	75 (39.7)	23 (12.2)
• Use only CATs to maintain my health.	10 (5.3)	71 (36.8)	71 (37.6)	22 (11.6)	15 (7.9)
• Use CATs with allopathic medicine to maintain my health and wellbeing.	11 (5.8)	89 (47.1)	63 (33.3)	14 (7.4)	12 (6.3)
• Disclose CATs usage to doctors.	7 (3.7)	48 (25.4)	64 (33.2)	51 (26.4)	19 (9.8)
• Think combination of CATs and allopathic medicine provided better seizure control.	10 (5.3)	50 (26.5)	83 (43.9)	33 (17.5)	13 (6.9)
• Advise others to use CATs.	5 (2.6)	46 (24.3)	82 (42.5)	38 (20.1)	18 (9.5)

CATs, complementary and alternative therapies.

### Adherence to AEDs

Over half of the participants had high adherence to AEDs (57.5%), but 23.7% had low adherence, which could put them at risk for complications. Common reasons reported for not adhering to their medication included forgetfulness (32.3%), missing doses while away from home (17.5%), and trouble remembering to take it regularly (14.4%). However, there were no significant association between CATs usage, knowledge, attitude and practice of CATs with the adherence levels to AEDs (*p* > 0.05) – Tables 4 and 5.

**Table 4.** Relationship between CATs usage and adherence to AEDs (*n* = 186).

Adherence level	CATs usage; n (%)		X^2^ (df)	*p*-value^a^
	Yes	No		
Low	23 (20.0)	21 (29.6)	2.303 (2)	0.316
Moderate	22 (19.1)	13 (18.3)		
High	70 (60.9)	37 (52.1)		

^a^ Chi-square test.

**Table 5.** Comparison of knowledge, attitude and practice of CATs scores and adherence status.

Variable (Range score)	Adherence level	Mean (SD)	F (df)	*p*-value^a^
Knowledge (0-4)	Low	2.29 (0.8)	0.523 (2)	0.588
	Moderate	2.03 (1.1)		
	High	2.13 (1.0)		
Attitude (5-40)	Low	24.37 (2.9)	0.695 (2)	0.500
	Moderate	25.23 (3.5)		
	High	24.67 (3.3)		
Practice (5-40)	Low	22.93 (5.0)	0.556 (2)	0.574
	Moderate	23.97 (5.1)		
	High	22.96 (5.2)		

^a^ One-way ANOVA test.

## Discussion

Medication adherence among patients with epilepsy is crucial for achieving optimal seizure control, as a consistent use of AEDs significantly impacts health outcomes (Singh et al., 2024). However, many patients struggle with adherence and turn to use CATs to increase their well-being. The present study found that 59.6% of participants reported using CATs, which aligns with the growing trend of CATs utilisation both in Malaysia and globally. This prevalence is comparable with a previous Malaysian study, which reported CATs usage among epilepsy patients ranging from 25% to 58% among their studied population (Farrukh et al., 2021; Koh et al., 2022). Besides, the usage rates of CATs among patients with epilepsy exhibit significant variability globally, from as low as 7.5% to as high as 73.3%, depending on cultural, socioeconomic and healthcare system differences (Al Asmi et al., 2013; Farrukh et al., 2021).

In the Middle East, CATs use is widespread, driven by strong religious and cultural beliefs. A study from Iran reported belief in CATs as high as 71%, with common practices including spiritual healing and herbal remedies (Asadi-Pooya et al., 2019). In Turkey, prevalence ranged from 20.8% to 82.0%, with motivations including enhancing medical treatment, reducing seizure frequency, and achieving seizure control (Gündüz et al., 2024; Özer et al., 2023). In South Korea, CATs use has reached 77.3%, often perceived as an affordable and natural alternative (Jeong et al., 2016). In contrast, Germany reported a lower prevalence of 13%, with users often integrating CATs alongside conventional medicine (Bosak & Słowik, 2019; Hartmann et al., 2016). Studies from United States of America (USA), showed usage around 27% to 30%, mostly among caregivers of children with epilepsy, driven by dissatisfaction with standard treatments and a preference for holistic care (Beattie et al., 2017; Kenney et al., 2016).

The most used CATs in this study were prayers (86.4%) and massage (78.8%), reflecting Malaysia’s strong religious and traditional cultural influences. This finding was similar to a previous study by Koh et al. (2022), demonstrating that prayers were the most preferred CATs modalities among participants due to the common part of religious identity. In addition, prayers and spiritual healing practices are widely accepted and often considered as a first-line remedy, particularly in rural or semi-urban areas (Can et al., 2024; Ghuloum et al., 2024). Globally, similar patterns have been observed in predominantly religious countries, where spiritual healing is perceived to complement conventional medicine (Çaksen, 2025; Hoosen et al., 2021).

Participants in this study demonstrated a moderate level of knowledge and a mildly positive attitude towards CATs. While many of them were familiar with certain CATs modalities and believed in their potential benefits, their understanding of safety, efficacy and potential interactions with medications was limited. The positive attitude may be shaped by cultural beliefs, personal or community experiences, social media and the perception that CATs are more natural and affordable alternatives (Aubin et al., 2023; Jifar et al., 2022). However, the lack of critical knowledge suggests that further education and guidance from healthcare professionals are needed to support patient decision making. Another notable finding was that only 29.1% of CATs users disclosed their use to doctors, highlighting a communication gap between patients and healthcare providers. Thisis consistent with previous studies, where patients often refrain from disclosing CATs use due to fear of disapproval, taboos or assumption that such therapies are irrelevant to discuss (Farrukh et al., 2021; Koh et al., 2022). These underscore the need for healthcare professionals to have a good communication strategy, routinely inquire about CATs use and provide information to ensure safe use of CATs.

Additionally, this present study revealed that 57.5% of participants demonstrated high adherence to AEDs, indicating that nearly half of the patients struggled with maintaining consistent medication intake. For instance, a study conducted among patients with epilepsy in Selangor, Malaysia by Farrukh et al. (2021) found adherence rates was 32%, while other global studies have reported adherence rates ranging from 50% to 80% (Singh et al., 2024). The most reported reasons for non-adherence to AEDs in this study were forgetfulness, missing doses while away from home and trouble in remembering to take medications regularly. These findings align with previous studies that highlight memory-related issues as a major barrier to medication adherence among people with epilepsy (Farrukh et al., 2021; Singh et al., 2024). These issues may be influenced by various factors, including busy daily routines, cognitive side effects of AEDs, or lack of structured medication-taking habits (Donahue et al., 2025; Singh et al., 2024). Thus, a multi-faceted approach including patient education, behavioural strategies and technological aids to support memory and routine. The healthcare providers also should frequently ask their patients about their use of CATs before prescribing any medicine to avoid undesirable adverse effects or drug interactions with CATs.

This study found no significant association between AEDs adherence level and the use of CATs, nor with participants’ knowledge, attitudes or practices related to CATs. This finding contrasts with some prior research that suggests CATs use may negatively influence medication adherence (Bosak & Słowik, 2019; Farrukh et al., 2021). It is probably due that the study population use CATs as a complementary to the AEDs rather than replace it. The individual health beliefs, patient-health professional communication, severity and duration of epilepsy could also affect the outcome (Jopowicz et al., 2024; Singh et al., 2024). Moreover, the moderate level of CATs knowledge and attitude observed among participants may have prevented reliance on CAT alone, reducing the likelihood of non-adherence to AEDs. These highlight the need for further exploration into other factors that may influence adherence in patients with epilepsy, such as social and psychological factors.

This study has several limitations. The cross-sectional design limits the ability to establish causal relationships between CATs usage and medication adherence. Besides, the use of self-reported questionnaires may introduce recall or social desirability bias especially regarding medication adherence and the use of CATs. Despite these limitations, this study is one of the few studies in Malaysia to explore the knowledge, attitude and practice towards CATs and their relationship with AEDs adherence. The inclusion of multiple hospitals enhances the representativeness of the data within the East Coast region. Moreover, the use of validated tools such as MALMAS adds credibility to the measurement of adherence levels. Due to the observational nature of this study, future research employing longitudinal designs or randomised controlled trials (RCTs) is necessary to more effectively assess causal links and intervention outcomes. Additional research is required to evaluate the safety, efficacy, and acceptability of CATs in epilepsy management, as well as to investigate the creation of integrative care models that correspond with local beliefs and practices.

Overall, the findings may potentially impact healthcare providers and policymakers, especially in incorporating culturally appropriate CATS into epilepsy management frameworks. Due to the nature of seizure, adverse effects of drugs and the tendency of patient to seek CATs, our findings emphasise the need for comprehensive & holistic management in epilepsy care.

## Conclusion

Generally, this study demonstrated over half of the patients with epilepsy reporting CATs usage, with many showing moderate knowledge and a positive attitude towards these therapies. Despite relatively high adherence to AEDs, no significant association was found between CATs usage, knowledge, attitude or practices and adherence levels. These findings suggest that CATs use does not necessarily interfere with AEDs adherence; however, continued education and patient-healthcare provider communication are important to ensure safe use of CATs. In order to explore how CATs could play a role in improving well-being, further research on CATs practice and safety evidence should be undertaken.

## Data Availability

Data sets and materials in this manuscript can be provided by the first author upon reasonable request.
